# Supplementary figures and images for: Genomic variability in Mexican chicken population using copy number variants
Source: BMC Genet. 2017 Jul 3;18:61. doi: 10.1186/s12863-017-0524-4 (PMC5496433; doi:10.1186/s12863-017-0524-4)

# Cluster dendrogram with AU/BP values (%)

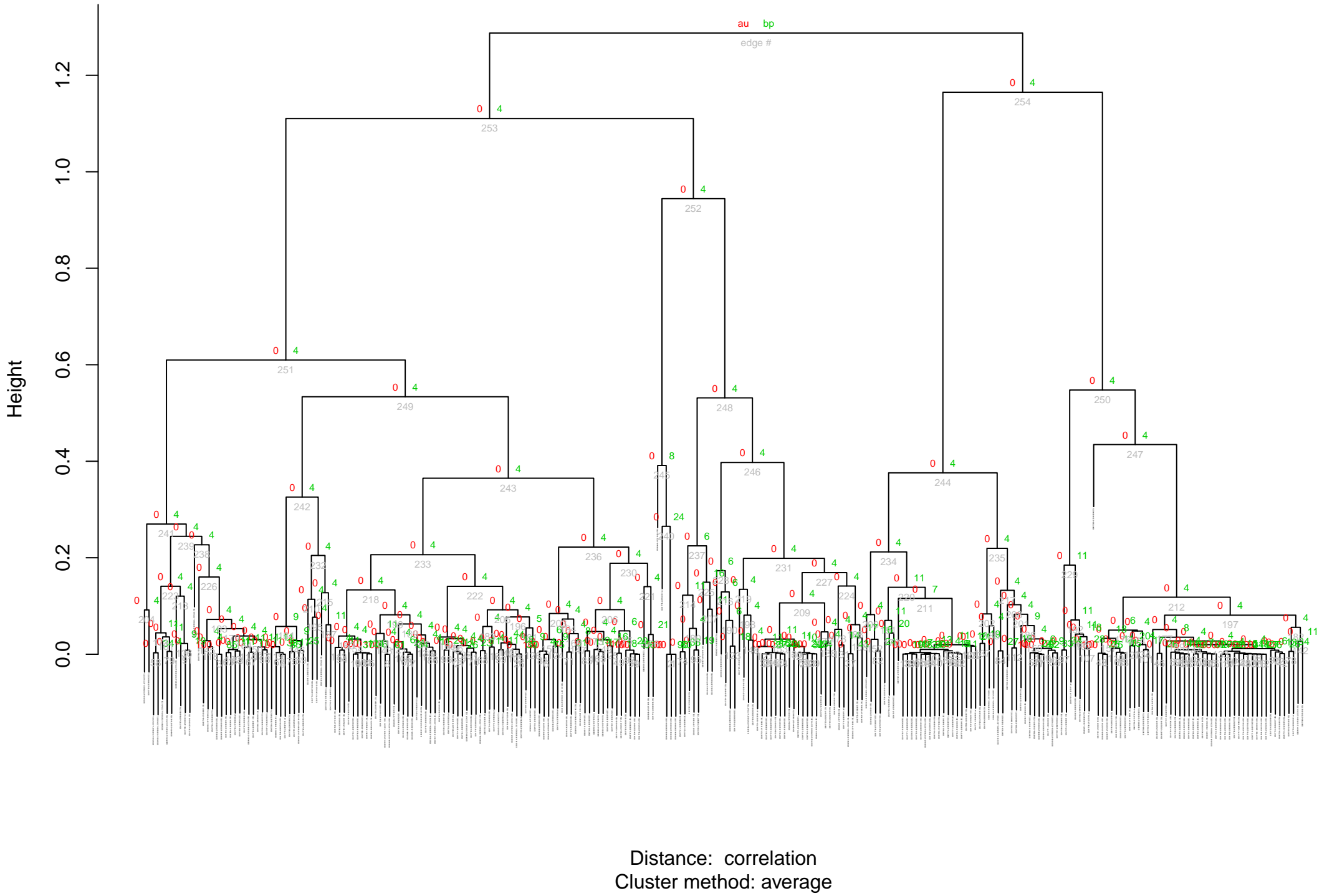

Supplement: Supplementary file 2 — Dendrogram of hierarchical cluster analysis of SNP genotypes (PDF 20 kb). [file 12863_2017_524_MOESM2_ESM.pdf]
